# Variation in reproductive effort, genetic diversity and mating systems across *Posidonia australis* seagrass meadows in Western Australia

**DOI:** 10.1093/aobpla/plaa038

**Published:** 2020-08-06

**Authors:** Elizabeth A Sinclair, Jane M Edgeloe, Janet M Anthony, John Statton, Martin F Breed, Gary A Kendrick

**Affiliations:** 1 School of Biological Sciences, University of Western Australia, Crawley, Western Australia, Australia; 2 Oceans Institute, University of Western Australia, Crawley, Western Australia, Australia; 3 Kings Park Science, Department of Biodiversity Conservation and Attractions, West Perth, Western Australia, Australia; 4 College of Science and Engineering, Flinders University, Adelaide, South Australia, Australia

**Keywords:** Environmental gradient, mating system, microsatellite DNA loci, monoecy, outcrossing rate, *Posidonia australis*, restoration, seed abortion

## Abstract

Populations at the edges of their geographical range tend to have lower genetic diversity, smaller effective population sizes and limited connectivity relative to centre of range populations. Range edge populations are also likely to be better adapted to more extreme conditions for future survival and resilience in warming environments. However, they may also be most at risk of extinction from changing climate. We compare reproductive and genetic data of the temperate seagrass, *Posidonia australis* on the west coast of Australia. Measures of reproductive effort (flowering and fruit production and seed to ovule ratios) and estimates of genetic diversity and mating patterns (nuclear microsatellite DNA loci) were used to assess sexual reproduction in northern range edge (low latitude, elevated salinities, Shark Bay World Heritage Site) and centre of range (mid-latitude, oceanic salinity, Perth metropolitan waters) meadows in Western Australia. Flower and fruit production were highly variable among meadows and there was no significant relationship between seed to ovule ratio and clonal diversity. However, Shark Bay meadows were two orders of magnitude less fecund than those in Perth metropolitan waters. Shark Bay meadows were characterized by significantly lower levels of genetic diversity and a mixed mating system relative to meadows in Perth metropolitan waters, which had high genetic diversity and a completely outcrossed mating system. The combination of reproductive and genetic data showed overall lower sexual productivity in Shark Bay meadows relative to Perth metropolitan waters. The mixed mating system is likely driven by a combination of local environmental conditions and pollen limitation. These results indicate that seagrass restoration in Shark Bay may benefit from sourcing plant material from multiple reproductive meadows to increase outcrossed pollen availability and seed production for natural recruitment.

## Teaser

Plant species including seagrasses reproduce using sexual (seeds) and asexual (vegetative) means which can vary across a species range. We compare sexual reproduction, genetic diversity and the mating system in *Posidonia australis* seagrass meadows from Western Australia. Flower and fruit production were two orders of magnitude lower in Shark Bay meadows which also had lower genetic diversity and a mixed mating system when compared with meadows in Perth metropolitan waters. These results indicate that seagrass restoration in Shark Bay may benefit from sourcing plant material from multiple reproductive meadows to increase outcrossed pollination and seed production for natural recruitment.

## Introduction

Populations at their geographic range edges tend to have smaller effective population sizes, reduced sexual reproduction and limited connectivity relative to centre of range populations (Eckert *et al.* 2008; Sexton *et al.* 2011). These patterns have been established through decades of theoretical and empirical studies in population genetics and integration with mating systems (reviewed in Charlesworth and Charlesworth 2017), although the pattern is less clear in marine species (e.g. Diekmann and Serrão 2012; Assis *et al.* 2013; Liggins *et al.* 2014). Renewed interest in understanding the drivers of biogeographic ranges has been reignited by the profound influence of climate change on distributional patterns of taxa and the ecosystems they inhabit (Chen *et al.* 2011; Nicastro *et al.* 2013; Wernberg *et al.* 2016). Distributional changes by species in the marine environment, particularly those inhabiting inshore coastal shelves, occur through sea level changes (Miller *et al.* 2011), as well as in response to changing climate (e.g. Pecl *et al.* 2017). The persistence of a species during these periods of change is ultimately influenced by available habitat and a species’ ability to respond to change. Some species’ ranges have not (yet) shifted, and their declines in demographic processes (e.g. survival or reproduction) are offset by increases in others (e.g. self-fertilization), potentially buffering populations from extinction (Sheth and Angert 2018). This may especially be the case for plant species with the ability to reproduce through sexual and asexual means (e.g. seagrasses), as adult plants may persist through extreme climate events over extended periods even when sexual reproduction fails.

Natural variation in traits, such as those associated with sexual reproduction, occur among populations across a species range; however, range edge populations may evolve physiological, morphological and life-history attributes that better attune them to warming environments (Levin 2012). These populations are also regarded as most threatened under climate change (Hampe and Petit 2005; Zardi *et al.* 2015). The extent to which individuals and populations have an outcrossed mating system can influence genetic structure, extent of gene flow, effective population size and expression of inbreeding depression (Barrett and Harder 2017). A recent meta-analysis by Whitehead *et al.* (2018) highlights the substantial variation in outcrossing rates across 105 species in which mating system analyses were obtained in more than three populations. Examination of mating systems in multiple populations provides an opportunity to assess links between specific influences—with a suggestion that abiotic pollination factors (e.g. wind, water) provide a greater opportunity for consistency in outcrossing rates (Whitehead *et al.* 2018). Goodwillie *et al.* (2005) reported elevated levels of environmental or genetic-based self-pollination also afford populations a measure of reproductive assurance, despite the genetic costs associated with inbreeding. We explore these hypotheses further in the marine environment where hydrophilous pollination is common.

Seagrasses are ancient marine flowering plants, of which most species complete their life cycle entirely underwater (Ackerman 1995). Globally, 24 % of species are classified as ‘threatened’ or ‘near-threatened’ on the IUCN’s Red List (Short *et al.* 2011), with the rate of decline continuing to increase due to anthropogenic activities including climate change (Orth *et al.* 2006; Waycott *et al.* 2009). Seagrasses play a central role in ecosystem services (Costanza *et al.* 1997; Lamb *et al.* 2017) and in mitigating climate change (Fourqurean *et al.* 2012; Duarte *et al.* 2013). Genetic data showing high outcrossing rates are common among monoecious species (summarized in Sinclair *et al.* 2014a), providing support for Ackerman’s hydrophilous pollination syndrome (Ackerman 2000). Here, we assessed reproductive (flower and seed production) and genetic data (diversity, population structure and mating system) for range edge and centre of range meadows of the Australian temperate seagrass, *Posidona australis*, a species with high seed dispersal capabilities (Kendrick *et al.* 2012, McMahon *et al.* 2014). We test the following hypotheses: (i) sexual reproduction is higher in Perth metropolitan waters than Shark Bay meadows; (ii) there is a shift from complete outcrossing in Perth metropolitan waters meadows to a mixed mating system in Shark Bay meadows; and (iii) the variance in outcrossing rates among *P. australis* ‘families’ within meadows is higher in mixed mating meadows than completely outcrossed meadows. The combination of reproductive and genetic data enables a more comprehensive understanding of seed production and the long-term implications for resilience and restoration of range edge seagrass meadows.

## Materials and Methods

### Study species

*Posidonia australis* is a perennial, marine angiosperm endemic to temperate Australian waters from Shark Bay at the northern range edge on the west coast to Wallis Lake on the east coast (Edgar 2000). It occurs in protected coastal waters and estuaries, just below the low water mark to 15 m water depth (Carruthers *et al.* 2007) and reproduces both vegetatively (clonal rhizome extension) and sexually (pollen and seed production). Thus, this long-lived species can persist through prolonged times of sexual reproductive failure. *Posidonia australis* is diploid (somatic chromosome number 2n = 20; Kuo *et al.* 1990). It has perfect or hermaphroditic flowers (den Hartog 1970), in which anthers mature and release pollen ahead of stigma receptivity (protandrous; McConchie and Knox 1989). Initiation of inflorescence development occurs in May (Austral autumn), with pollination occurring in July/August (Austral winter) and fruit release in November–January (Austral spring–summer). Timing of fruit release varies with latitude; fruits ripen 1 month earlier in Shark Bay (25–26°S) than Perth metropolitan waters (32°S). Inflorescences are positioned above the leaf canopy, with up to 20 fruits being produced per inflorescence. Flower and seed production are temporally and spatially variable across the species range, but typically annual and prolific in Perth metropolitan waters (Cambridge and Hocking 1997). Flowering meadows in Shark Bay cover a few km^2^ compared with 10s to 100s km^2^ in Perth metropolitan waters.

### Field sites

**Figure 1.**
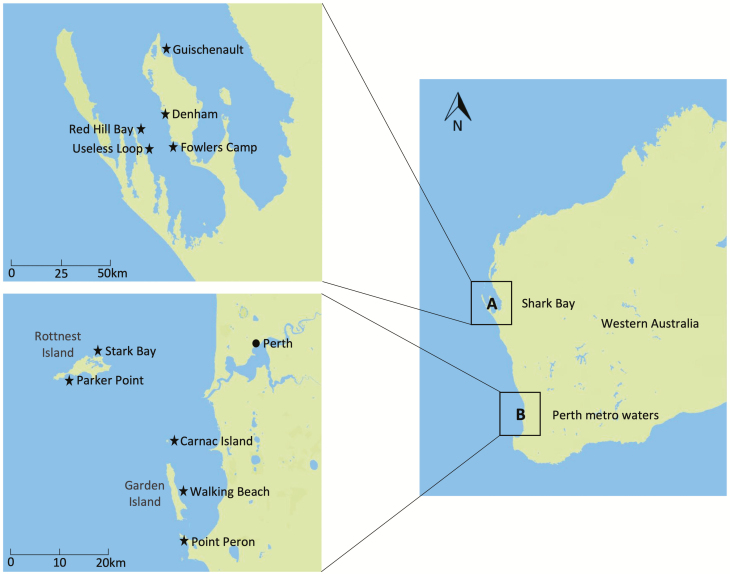
Map of Western Australia showing the location of sampled meadows in Shark Bay, inset A: Guischenault, Red Hill Bay, Denham, Useless Loop, Fowlers Camp, and meadows in Perth metropolitan waters, inset B: Stark Bay and Parker Point, Rottnest Island, Carnac Island, Walking Beach, Garden Island, Point Peron.

Flowering meadows were sampled from northern range edge meadows in Shark Bay and mid-latitude meadows in Perth metropolitan waters (Fig. 1; Table 1). All sampled meadows were situated in shallow waters (<3 m) on the broad continental shelf off the Western Australian coastline. Palaeo sea level records from Western Australia, including tubeworm data from Rottnest Island (Playford 1988; Baker *et al.* 2005), indicate that sea level was within 2 m of its modern level by mid-Holocene (~7100 ± 70 cal. years BP); thus, seagrass meadows are likely to have been broadly stable since that time.

**Table 1.** Location and characteristics for *Posidonia australis* meadows.

Location (north–south)	Abbrev.	Latitude (S)	Longitude (E)	Salinity (PSU)	Depth (m)	Meadow characteristics	Hydrodynamics
Shark Bay (northern range edge):							
Guischenault	GU	−25.61895	113.58918	36–38	<2	Expansive meadow	Large tides at time of pollen release, water tends to spill off the banks
Red Hill Bay	HP	−26.03051	113.37399	36–38	<2	Fringing seagrass, highly fragmented	Strong tidal movement
Fowlers Camp	FC	−26.10549	113.61285	>40	<2	Fringing seagrass near expansive meadow	Large tides at time of pollen release, sheltered from waves
Perth metropolitan waters (centre of range):							
Stark Bay, Rottnest Island	RST	−32.00604	115.48488	Oceanic	<3	Expansive meadow mixed with rocky reef	Exposed to waves from the north
Parker Point, Rottnest Island	RPP	−32.02242	115.53092	Oceanic	<2	Fringing seagrass, highly fragmented	Sheltered with weak current
Carnac Island, Cockburn Sound	CI	−32.12040	115.66547	Oceanic	<3	Fringing seagrass, fragmented	Sheltered meadow with strong water movement from swell and wind refracting around the island

Shark Bay, to the north, was formed by a marine transgression ~7000–8000 years ago (Bufarale and Collins 2015). Radiocarbon dating of sediment cores indicates seagrass has been present in the Bay throughout the Holocene (not earlier than 8.5–8.0 ka BP, Bufarale and Collins 2015; 3000 years, Serrano *et al.* 2016). Shark Bay is an inverse estuary with a permanent salinity gradient—from oceanic in the north to hypersaline conditions in the southern reaches (35–70 practical salinity unit (PSU)). The salinity gradient has been maintained since the last sea level adjustments (~4500 years ago) by the formation of seagrass-dominated sills and banks that have restricted water movement and nutrients (Fraser *et al.* 2012; Bufarale and Collins 2015). Shark Bay is recognized as a UNESCO World Heritage Site (WHS) for its unique, highly biodiverse ecosystem at the interface of warm tropical and southern temperate zone ecosystems and home to the largest reported seagrass meadows in the world (Walker *et al.* 1988; Kendrick *et al.* 2012). The mostly pristine nature and legal protection afforded to the marine environments around Rottnest and Carnac Islands and Shark Bay WHS, within which *P. australis* meadows inhabit, provide an opportunity to understand contemporary processes relatively free from localized anthropogenic threats.

### Sexual reproduction

Flower and fruit production were measured *in situ* for two meadows in Shark Bay (Guischenault, Red Hill Bay) and two meadows in Perth metropolitan waters (Stark Bay and Parker Point, Rottnest Island) during Spring 2016. Inflorescence density was estimated by using five replicate 10 m × 1 m (10 m^2^) belt transects. Flower and fruit production per inflorescence were estimated from the random collection of 12 inflorescences from transects at each site. Inflorescences consisted of a stem (petiole) bearing several spikes (3–12) with 3–5 hermaphrodite flowers. Following successful pollination, fruit development takes ~12 weeks. The number of fully developed fruit, undeveloped fruit and remains of flowers that had not been pollinated were counted on each inflorescence spike. The total number of flowers per inflorescence was derived from the sum of all fruit, undeveloped fruit and remains of flowers. Floral (number of flowers per m^2^) and fruit density (number of fruits per m^2^) were determined before fruit release. Production at Fowlers Camp was estimated based on fruit scars, as fruit had released prior to sampling. Seed to ovule ratio was determined from the total number of mature fruit (1 fruit = 1 seed) divided by the total numbers of flowers (1 flower = 1 ovule). The floral and fruit density data and seed to ovule ratios were assessed for heteroscedasticity and normality. A Tukey Ladder of Powers approach (Tukey 1977) was used to power transform the data to maximize normality of residuals. The normality of residuals was visualized and assessed with a Shapiro–Wilk tests for normality. A one-way ANOVA (meadow as fixed factor) and Tukey’s HSD test were performed for multiple comparisons of means between sites and regions, with a 95 % confidence level.

### Genetic sampling, DNA extraction and genotyping

**Figure 2.**
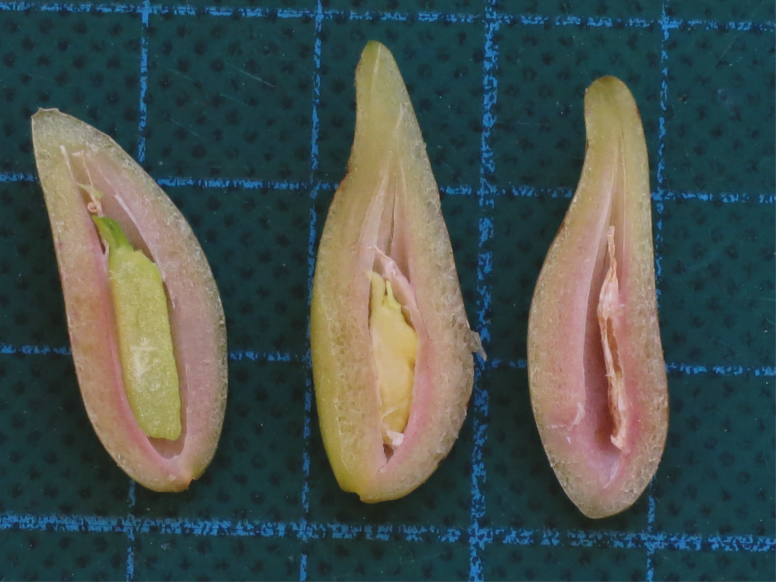
Transverse section of mature *Posidonia* fruit showing (left to right) viable, ‘mutant’ and aborted embryos. Scale: one square = 1 cm. Photo by E. A. Sinclair.

Opportunistic collections of adult shoots and associated inflorescences were made on SCUBA between 2014 and 2018 at three meadows in Shark Bay (Guischenault, Red Hill Bay, Fowlers Camp) and three meadows in Perth metropolitan waters (Stark Bay and Parker Point, Rottnest Island, and Carnac Island, Cockburn Sound; Fig. 1). Approximately 30 individual maternal shoots were collected from each meadow just prior to fruit dehiscence (with the exception of Fowlers Camp) using methods described in Sinclair *et al.* (2014a). A visual assessment of seed viability (viable, mutant or aborted; Fig. 2) was performed prior to DNA extraction. Aborted embryos were discarded as there was not sufficient tissue to genotype.

DNA was extracted from shoot meristem and embryos using methods previously described (Sinclair *et al.* 2009, 2014a). Seven polymorphic microsatellite loci were used to generate multilocus genotypes (MLGs) using two multiplex mixes containing 5.2 µL of 2× Multimix and 1.98 µL of 5× Q sol (Type-It Microsatellite PCR kit; Qiagen, Hinden, Germany), 1.0 µL of primer mix (PM) and 2.0 µL of 5–10 ng DNA in a 10 µL reaction. Primer mix 1 contained the primers *Pa*A1, *PaA*105, *Pa*A120; primer mix 2 contained *Pa*B6, *Pa*B8, *Pa*B112, *Pa*D113. Forward primers were fluorescently labelled (FAM, VIC or PET; **see****Supporting Information—Table S1**) and microsatellite regions were amplified for all individuals by PCR using a Veriti thermocycler (Thermo Fisher Scientific, Waltham, MA, USA) using the following PCR conditions: an initial 1-min denaturation at 95 °C, 35 cycles of 94 °C for 10 s, 60 °C for 30 s and 72 °C for 45 s followed by a final extension of 15 min at 60 °C. Electrophoresis was run on an ABI 3500 sequencer (Life Technologies) with size standard LIZ. Allele sizes were scored using Geneious version 7.1 (Biomatters Ltd, Auckland, New Zealand). Replicate PCRs were performed to ensure the accuracy of the final data set. There was no evidence of linkage disequilibrium or null alleles at these loci based on previously obtained diploid genotypes (Sinclair *et al.* 2009, 2014a, b, 2016a); however, we ran all new diploid data (see below) through Micro-Checker v2.2.3 to assess for the presence of scoring error due to stuttering, large allele dropout or null alleles (van Oosterhout *et al.* 2004; http://www.nrp.ac.uk/nrp-strategic-alliances/elsa/software/microchecker/).

### Genetic diversity, clonal diversity and genetic structure

Tri-allelic genotypes were observed in six out of the seven loci that typically give di-allelic genotypes. Allele frequencies were therefore calculated using GENODIVE version 2.0b27 (Meirmans and Van Tienderen 2004), which handles genotypes with more than two alleles per locus and permits mixed (diploid and triple allelic) genotypes to be included within the same analyses. The summary statistics calculated on the complete data set were: total number of alleles (*N*_a_), private alleles (*p*_[i]_), mean number of alleles (Num), effective number of alleles (Eff Num), observed (*H*_o_) and expected (*H*_s_) heterozygosity within sampled meadows and seed ‘populations’. The ‘*Assign clones*’ option was used to identify unique clones or MLGs and their frequency due to the high chance of sampling multiple flowering ramets from the same plant within a meadow. We implemented a stepwise mutation model (SMM), with a threshold of 1. This was used due the unusually large number of MLGs differing by a single allele at Carnac Island (see Sinclair *et al.* 2014b). Clonal richness (*R* = (*G* − 1)/(*N* − 1)), where *G* = number of MLGs, and *N* = number of samples, was estimated for each meadow (Dorken and Eckert 2001) and clonal evenness (ED), a measure of abundance of MLGs. Deviations from Hardy–Weinberg equilibrium, described as an inbreeding coefficient (*G*_is_), were calculated based on MLGs with 10 000 permutations, with positive values indicating a heterozygote deficit and negative values indicating a heterozygote excess. We conducted a *t*-test to determine whether there was a significant difference between clonal diversity in embryo ‘populations’ in Shark Bay and Perth metropolitan waters. Genetic differentiation (*F*_ST_) was generated for all pairs of ‘populations’ using the complete data set, with significance between populations assessed using with 10 000 permutations.

### Mating system

Many of the standard population genetic tools have been developed for diploid data sets and therefore not feasible for polyploid or mixed data sets (Dufresne *et al.* 2014). In the absence of any evidence of polyploidy in *P. australis* (Sinclair *et al.* 2016b), individuals containing tri-allelic genotypes were reduced to diploid genotypes to enable estimation of mating system parameters using the software program MLTR v.3.4 (Ritland 2002; http://kermitzii.com/softwares/). Alleles that were not detected in a homozygous form, or were rare (where *f* < 0.05), were removed. Identical, commonly occurring tri-allelic genotypes were reduced to the same diploid genotype so as not to alter the total number of MLGs. All genotypes were manually checked to ensure each embryo contained at least one maternal allele. We acknowledge that this may introduce bias through an artificial increase in estimated selfing rates. Mating system parameters were estimated for each meadow, as well as by families within each meadow, using a maximum likelihood approach. These estimates were based on the multilocus mixed-mating model that assumes plants are randomly mating and the level of outcrossing is inversely proportional to the level of selfing (Shaw *et al.* 1981; Ritland 2002). The program was used to derive both single (*t*_s_) and multilocus (*t*_m_) outcrossing rates, biparental inbreeding (*t*_m_*–t*_s_, proportion of embryos due to mating between closely related parents) and multilocus correlation of *P* (proportion of full siblings) (Ritland 2002). Outcrossed embryos were unambiguously identified by the presence of a non-maternal allele. The level of inbreeding among maternal plants was characterized by the inbreeding coefficient (*f*), where a positive value indicates an excess of homozygotes and a negative value indicates an excess of heterozygotes as a result of outcrossing, as compared to expectations under Hardy–Weinberg equilibrium. Standard errors for all estimates were derived using a bootstrap method with 1000 bootstraps. The effective number of pollen donors (*N*_ep(w)_) per inflorescence (= family) was estimated by taking the inverse of the *r*_p_ (Smouse *et al.* 2001). We conducted a *t*-test to determine whether there was a significant difference between outcrossing rates in meadows from Shark Bay and Perth metropolitan waters.

## Results

### Sexual reproduction

**Figure 3.**
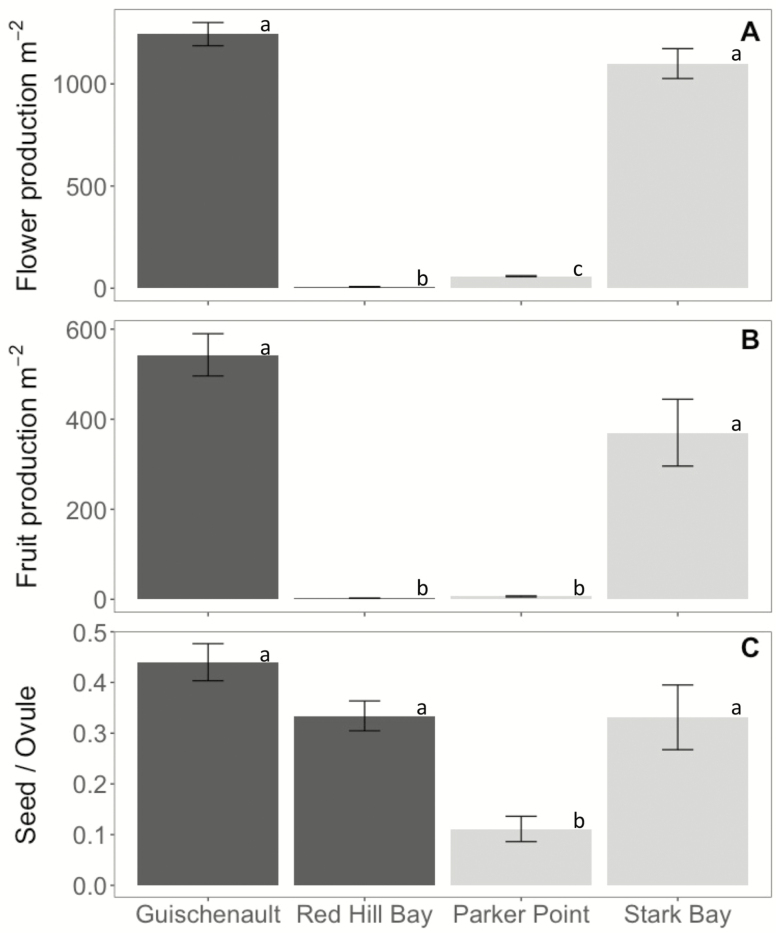
Sexual reproductive output between four sampled *P. australis* meadows, as measured by (A) flower production, (B) fruit production and (C) seed to ovule ratio. The letters above the graphs (a–c) represent significant statistical outcomes from a one-way ANOVA and Tukey’s HSD test, *P* < 0.0001. Shark Bay meadows are Guischenault and Red Hill Bay, Perth metropolitan waters are Stark Bay and Parker Point, Rottnest Island.

There was a significant difference in sexual reproduction among meadows, as measured by flower production, fruit production and seed to ovule ratio (Fig. 3A–C). Flower production was very high (>1000 m^−2^) at Guischenault and Stark Bay, with no statistically significant differences in densities recorded (Fig. 3A). They were both significantly higher than Parker Point (<50 m^2^) and Red Hill Bay (<10 m^−2^) (Tukey’s HSD test, *P* < 0.0001). A similar pattern was observed in fruit production (Fig. 3B). The only difference being that Parker Point and Red Hill Bay had an extremely low number of fruit (<10 m^−2^) and they were not significantly different from each other. Parker Point had a significantly lower seed to ovule ratio than the other three sites (Tukey’s HSD test, *P* < 0.0001). Thirty-two to 45 % of all ovules produced seed in the other three sites and differences in seed to ovule ratio were not significant (Fig. 3C).

### Genetic diversity, clonal diversity and genetic structure

There was no systematic evidence of scoring error due to stuttering, large allele dropout or null alleles across loci and populations. However, the presence of null alleles due to high homozygosity was suggested in embryo populations from Guischenault (three loci) and Red Hill Bay (one locus). Overall genetic diversity estimates were higher in Perth metropolitan waters relative to Shark Bay meadows (Table 2; **see****Supporting Information—Table S2**). Clonal diversity among maternal shoots was similar (*R* = 0.17–0.41; Table 2), with the exception of Carnac Island which was high (*R* = 0.94). Centre of range embryo populations had significantly higher clonal diversity (*R* = 0.66–0.96) relative to embryos from range edge meadows (*R* = 0.30, 0.26) (*t*-test: −4.66, *P*-value = 0.009). Clonal evenness was lower in embryo populations than meadows for range edge meadows, with 47.8 % (45/94) of embryos from Guischenault sharing the same MLG. The pattern was reversed in centre of range meadows, with the exception of Carnac Island (Table 2). One to three private alleles were detected in all embryo populations. Significant deviations from Hardy–Weinberg equilibrium were detected in most maternal and embryo populations, likely as a result of high clonality due to selective sampling for reproductive shoots.

**Table 2.** Summary of genetic diversity and clonal indices for sampled *P. australis* shoots and embryos; *N* = number of shoot samples; MLG = multilocus genotypes; *R* = number of unique clonal richness; ED = clonal evenness; 3× = number of samples with an additional allele in at least one locus; *N*_a_ = total number of alleles; *p*_[i]_ = private alleles; Num = mean number of alleles; Eff Num = effective number of alleles; *H*_o_ = observed heterozygosity; *H*_s_ = expected heterozygosity; *G*_is_ = inbreeding coefficient; *significant deviation from Hardy–Weinberg equilibrium at *P* < 0.05.

Sampling location and type	Abbrev.	Coll. year	*N*	MLG	*R*	ED	3×	*N*_a_	*p*_[i]_	Num	Eff Num	*H*_o_ (%)	*H*_s_ (%)	*G*_is_
Shark Bay (northern range edge):														
Guischenault														
Shoots (maternal)	GU	2014	28	11	0.37	0.54	6	18	1	2.57	2.57	17.9	16.9	−0.058*
Embryos (offspring)	GUe	2014	123	37	0.30	0.17	47	21	2	3.00	1.39	16.7	24.1	0.307*
Red Hill Bay														
Shoots (maternal)	HP	2016	15	4	0.21	0.62	0	11	0	1.57	1.37	35.7	20.5	−0.722*
Embryos (offspring)	HPe	2016	94	25	0.26	0.43	2	15	1	2.14	1.36	19.3	19.3	−0.001
Fowlers Camp (maternal)	FC	2018	27	6	0.19	0.39	27	20	2	2.86	1.92	47.1	34.6	−0.360*
Perth metropolitan waters (centre of range):														
Stark Bay, Rottnest Isl.														
Shoots (maternal)	RST	2014	28	12	0.41	0.59	7	24	0	3.43	2.48	59.7	50.6	−0.180*
Embryos (offspring)	RSTe	2014	106	102	0.96	0.97	26	35	3	5.00	2.82	57.1	53.5	−0.069*
Parker Point, Rottnest Isl.														
Shoots (maternal)	RPP	2014	30	6	0.17	0.45	0	21	0	3.00	1.58	36.2	31.3	−0.156*
Embryos (offspring)	RPPe	2014	144	115	0.80	0.83	0	27	1	3.86	1.82	50.1	41.8	−0.198*
Carnac Island														
Shoots (maternal)	CI	2014	32	30	0.94	0.95	1	29	0	4.14	1.63	30.4	29.6	−0.025
Embryos (offspring)	CIe	2014	274	182	0.66	0.46	9	36	2	5.14	1.59	29.8	28.9	−0.031*

Shoot samples with identical MLGs within a meadow were assumed to belong to the same flowering genet (or clone), while shared embryo MLGs were likely a result of low genetic diversity, self-pollination and/or apomixis. Twenty-six MLGs were shared among shoot and embryo ‘populations’ within Guischenault and Red Hill Bay. A single MLG was shared among two embryos from Guischenault and shoots from Red Hill Bay (*n* = 1), Useless Loop (*n* = 2) and Fowlers Camp (*n* = 17). Overall, there was significant genetic differentiation among meadows (adult shoots *F*_ST_ = 0.149, *P* < 0.001), with no significant differentiation between maternal shoot and embryo ‘populations’ from Red Hill Bay, Stark Bay and Carnac Island **[see****Supporting Information—Table S3****]**. Maternal shoot and embryo ‘populations’ were weakly differentiated at Guischenault and Parker Point.

**Figure 4.**
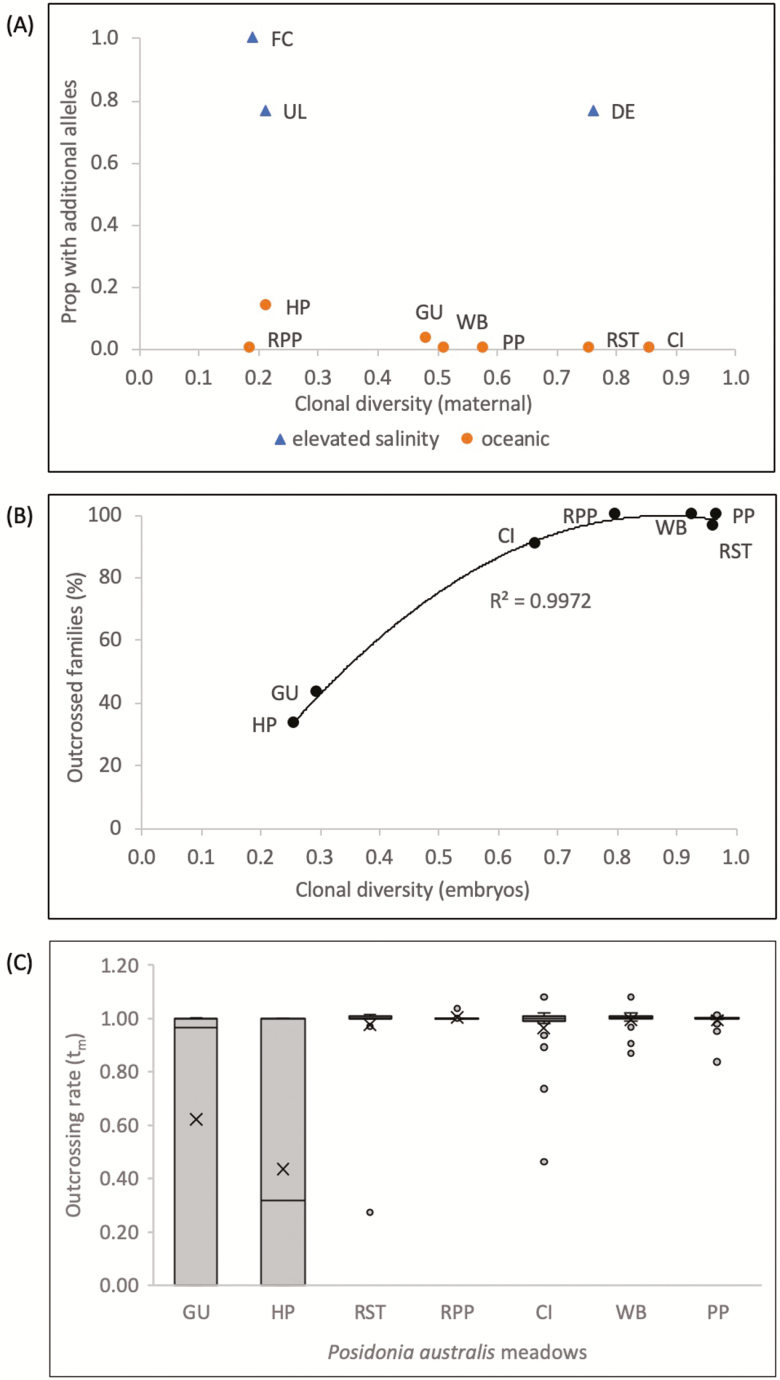
Genetic diversity: relationship between (A) clonal diversity and proportion of samples with 3× alleles within a meadow relative to salinity, (B) clonal diversity in embryos and percentage of outcrossed families by meadow and (C) variance in outcrossing rates (*t*_m_) among *P. australis* ‘families’ by seagrass meadow. The box corresponds to the lower and upper quartiles (25 and 75th percentiles), internal horizontal bars indicate the median and vertical whiskers extend to the lowest and highest values no further than 1.5 interquartile range. Points outlying this are represented as dots. Abbreviations for range edge meadows in Shark Bay are Guischenault (GU), Red Hill Bay (HP), Denham (DE), Useless Loop (UL), Fowlers Camp (FC) and Perth metropolitan waters are Stark Bay (RST) and Parker Point (RPP) on Rottnest Island, Carnac Island (CI), Walking Beach, Garden Island (WB), Point Peron (PP).

Additional alleles (tri-allelic genotypes) were present in at least one locus for every shoot genotype at Fowlers Camp, contributing to elevated observed heterozygosity (*H*_o_) relative to Guischenault. Tri-allelic genotypes were also observed in some embryos (Table 2). The proportion of samples with additional alleles is much higher in Shark Bay meadows with elevated salinities, regardless of clonal diversity (Fig. 4A).

No significant relationship was observed between seed to ovule ratios and shoot clonal diversity using maternal genotypes and previously collected population genetics data (summarized in Table 4) from meadows on the west coast of Australia (Pearson *r* (maternal) = 0.763, *P* = 0.133; Pearson *r* (population): 0.597, *P* = 0.287).

### Mating system

Mating system analyses were conducted on samples from five meadows which produced viable embryos. Complete outcrossing was seen in the three meadows from Perth metropolitan waters (Stark Bay, Parker Point, Carnac Island; Table 3). Mixed mating (i.e. self- and cross-fertilized embryos) was observed in the two Shark Bay meadows that produced viable embryos, with multilocus outcrossing rates of 0.50 at Guischenault and 0.57 at Red Hill Bay (Table 3). There was a significant difference in outcrossing rates between meadows in Shark Bay and Perth metropolitan waters (*t*-test: −7.75, *P*-value < 0.01). Correlated paternity (or proportion of full siblings) was much higher in Shark Bay meadows (*r*_p_): range edge = 0.90, 0.93 than Perth metropolitan waters *=* 0.11–0.21, indicating a much higher effective number of pollen donors per inflorescence for Perth metropolitan waters (4.8–8.8) compared with Shark Bay (~1.0) meadows. Carnac Island was the exception, with the highest proportion of full sibs from all sampled meadows in Perth metropolitan waters (0.68). Outcrossing rates within individual families were much more variable in Shark Bay meadows than Perth metropolitan waters meadows (Table 3), with a significant correlation between clonal diversity in embryos and percentage of outcrossed families by meadow, regardless of location (Fig. 4B, Pearson *r* = 0.956, *P*-value < 0.001). A visual inspection of Fig. 4C shows a much larger variance in family outcrossing rates in meadows with mixed mating systems in Shark Bay relative to completely outcrossed meadows across Perth metropolitan waters.

**Table 3.** Mating system parameters for *Posidonia australis* meadows and by ‘families’ within meadows. Values are based on genotypes for seven microsatellite loci. *See Fig. 2.

Parameter	Shark Bay meadows		Perth metropolitan waters		
	GU (±SD)	HP (±SD)	RST (±SD)	RPP (±SD)	CI (±SD)
No. mature embryos genotyped/infructescence	2–8	2–10	2–8	3–8	3–14
Mean family size	4.4 (±1.6)	6.3 (±2.4)	3.8 (±1.3)	4.8 (±1.4)	8.6 (±2.5)
Total number of aborted embryos	9	15 (+21 mutant*)	1	2	5
Percentage of aborted embryos	0.40 %	13.60 %	0.08 %	1.30 %	1.70 %
Number of families genotyped	28	15	28	30	32
Number of embryos genotyped	123	94	106	144	274
Mating system parameters by meadow:					
Parental inbreeding *f*	0.19 (±0.15)	−0.20 (±0.01)	−0.09 (±0.02)	−0.11 (±0.06)	−0.20 (±0.05)
Multilocus outcrossing rate *t*_m_	0.50 (± 0.09)	0.57 (± 0.26)	1.03 (±0.02)	1.20 (±0.03)	1.06 (±0.04)
Single-locus outcrossing rate *t*_s_	0.54 (±0.10)	0.59 (±0.27)	1.13 (±0.03)	1.20 (±0.00)	1.04 (±0.04)
Biparental inbreeding *t*_m_*–t*_s_	−0.04 (±0.03)	−0.02 (±0.04)	−0.10 (±0.04)	0.00 (±0.04)	0.02 (±0.04)
Multilocus correlation of *P* within genets *r*_p(w)_	0.90 (±0.19)	−0.93 (±0.33)	0.11 (±0.13)	0.21 (±0.06)	0.68 (±0.02)
Effective number of pollen donors *N*_ep(w)_	1.1	1.0	8.8	4.8	1.5
Mating system parameters within family:					
Percentage (%) outcrossed families	43.3	33.3	96.4	100.0	90.6
Single-locus outcrossing rate—range *t*_s_ (±SE)	−0.41–1.99 (±0.25)	−0.72–2.40 (±0.40)	0.10–1.73 (±0.16)	1.04–1.93 (±0.16)	0.38–1.32 (±0.11)
Multilocus outcrossing rate—range *t*_m_ (±SE)	−0.41–1.49 (±0.16)	−0.75–2.16 (±0.48)	0.27–1.06 (±0.00)	1.00–1.10 (±0.00)	0.46–1.38 (±0.04)

## Discussion

*Posidonia australis* meadows were completely outcrossed in Perth metropolitan waters while range edge meadows in Shark Bay had a mixed mating system. We also observed lower levels of genetic diversity and higher clonality in meadows within Shark Bay relative to Perth metropolitan waters, which together support our population genetic hypotheses, despite limited replication to distinguish between an edge effect and the potentially confounding effect of elevated salinity. Low levels of genetic diversity within individual meadows with low to no sexual reproduction were also reported in several northern range edge *P. australis* meadows on the east coast of Australia (Evans *et al.* 2014). However, the considerable diversity retained across *P. australis* meadows in Shark Bay may be the result of genetic connectivity over larger spatial areas and longer time periods, as well as additional alleles. A regional assessment of eelgrass, *Zostera marina*, showed that genetic diversity was retained across multiple (southern) range edge meadows relative to within individual meadows which had small effective population size, reduced habitat area, low sexual reproduction and gene flow (Diekmann and Serrão 2012). Marine seascape patterns are often complex, with temperature, oceanography and geography showing equal prevalence of influence on spatial genetic patterns (reviewed in Selkoe *et al.* 2016).

Our hypotheses appear less well supported by productivity data on flowering and seed production and ratio of seed set to flowering (seed to ovule ratio). Sampling was however focussed on seed-producing meadows to obtain data on outcrossing rates. The spatial extent of the Shark Bay meadows was two orders of magnitude lower than Perth metropolitan waters where there are 10s to 100s km of reproductively fecund meadows. There are many more meadows in Shark Bay that have low densities of flowers, with no viable seeds being produced (see Kendrick *et al.* 2019).

Our combined *P. australis* data for seven meadows (Sinclair *et al.* 2014a; this study) are consistent with patterns observed in terrestrial plant species, regardless of pollination method whereby range edge populations tend to have mixed mating. The potentially false increases to selfing rates introduced by reducing tri- to bi-allelic genotypes to estimate outcrossing rates are unlikely to account for such significant differences observed. The high frequency of additional alleles (tri-allelic genotypes) present in three non-reproductive Shark Bay meadows genotyped (Table 4, Denham (DE), Fowlers Camp (FC) and Useless Loop (UL)) contributed to elevated heterozygosity (*H*_o_) relative to Guischenault (GU), which was the most fecund meadow. Alternative hypotheses proposed to explain the presence of additional alleles across multiple meadows include ancient hybridization, putative aneuploidy and somatic mutations leading to genetic mosaicism, all of which have been reported in seagrasses (Reusch and Boström 2011; Sinclair *et al.* 2019; Digiantonio *et al.* 2020). The accumulation of somatic mutations (leading to genetic mosaicism) could explain the additional alleles; however, it is unlikely to account for the high frequency and widespread observations across Shark Bay and beyond. The more widespread occurrence of additional alleles suggests they made be a legacy of hybridization event(s), whereby a diploid F1 hybrid plant is fertile and able to backcross to a parental species (Sinclair *et al.* 2016b). Additional alleles in the backcross hybrid may be caused by unreduced (diploid) pollen combining with the haploid pollen from either parental species. These backcross hybrids persist through vegetative growth, but are probably sterile, and consistent with reduced or complete failure to produce viable seed in these meadows. Such an explanation is unlikely in the absence of polyploidy and/or whole-genome duplication (see ploidy cycling in Wendel 2015). Additional research with appropriately designed sampling using genomic approaches may determine the true origin of additional alleles in the future, as demonstrated in another seagrass genus, *Zostera* (Yu *et al.* 2020).

**Table 4.** Summary of all genetic data from Shark Bay meadows and mating system studies for *Posidonia australis* meadows. Meadow abbrev. = random shoot sample from meadow; meadow abbrev. with ‘m’ denotes maternal genotypes only; meadow abbrev. with ‘e’ denotes embryo genotypes only; *N* = number of samples; MLG = number of unique multilocus genotypes; *R* = clonal richness; *H*_o_ = observed heterozygosity; 3× = number of samples with an additional allele in at least one locus; *fruit were not collected, so it was not possible to determine whether the embryos were viable.

Meadow abbrev.	Salinity (PSU)	Coll. year	*N*	MLG	*R*	*H*_o_ (%)	3×	Fruit	Seed: ovule	Outcrossing rate	Pollen donors	Prop of full sibs	Source
Shark Bay (northern range edge):													
GU	35–38	2012	30	15	0.48	25.9	1						Unpubl. data
GUm		2014	28	11	0.37	17.9	6						This paper
GUe		2014	123	37	0.30	16.7	47	Yes	0.45	50 %	1.1	0.90	This paper
HP	35–38	2016	29	7	0.21	42.9	4						This paper
HPm		2016	15	3	0.14	35.7	0						This paper
HPe		2016	94	25	0.26	19.3	2	Yes	0.32	57 %	1.0	0.93	This paper
UL	>38	2011	37	13	0.33	62.9	29	Yes	–	–	–	–	Sinclair *et al.* (2016b)
DE	>38	2012	30	16	0.52	55.2	23	–	–	–	–	–	Unpubl. data
FC	>40	2018	27	6	0.19	47.1	27	Yes*	0.07	–	–	–	This paper
Perth metropolitan waters (centre of range):													
RST	Oceanic	2015	50	38	0.76	54.1	0						Unpubl. data
RSTm		2014	28	12	0.41	59.7	7						This paper
RSTe		2014	106	102	0.96	57.1	26	Yes	0.32	100 %	8.8	0.11	This paper
RPP	Oceanic	2009	49	10	0.19	54.3	0						Sinclair *et al.* (2014b)
RPPm		2014	30	6	0.17	36.2	0						This paper
RPPe		2014	144	115	0.80	50.1	0	Yes	0.10	100 %	4.8	0.21	This paper
CI	Oceanic	2009	50	43	0.86	30.6	0						Sinclair *et al.* (2014b)
CIm		2014	32	30	0.94	30.4	1						This paper
CIe		2014	274	182	0.66	29.8	9	Yes	–	100 %	1.5	0.68	This paper
PP	Oceanic	2010	46	27	0.58	54.8	0						Sinclair *et al.* (2014b)
PPm		2010	32	15	0.45	0.49	0						Sinclair *et al.* (2014a)
PPe		2010	213	206	0.97	0.51	0	Yes	–	100 %	11.1	0.09	Sinclair *et al.* (2014a)
WB	Oceanic	2010	48	25	0.51	52.0	0						Sinclair *et al.* (2014b)
WBm		2010	34	22	0.64	0.46	0						Sinclair *et al.* (2014a)
WBe		2010	208	193	0.93	0.47	0	Yes	–	100 %	6.6	0.15	Sinclair *et al.* (2014a)

### Role of the local environment on sexual reproduction

Understanding the relative influence of geographic location and environmental conditions on sexual reproduction is challenging. The relationship between reproductive output and genetic diversity measures may also be affected by local environmental conditions (e.g. hydrology and salinity). The Shark Bay meadows had fewer reproductive clones and a mixed mating system, although flower and fruit production and seed to ovule ratios can be comparable to meadows in Perth metropolitan waters. The Guischenault meadow has some of the highest recorded flowering and fruit densities in *P. australis* with a high seed to ovule ratio, yet overall levels of genetic diversity were low in maternal plants and embryos, with only 50 % outcrossing. In contrast, Red Hill Bay has some of the lowest recorded flowering and fruit densities in *P. australis* with very low genetic and clonal diversity (similar to Parker Point) yet has a similarly high seed to ovule ratio and mixed mating. Both these reproductive range edge meadows have high water movement (i.e. strongly tidal, linear movement) at close to oceanic salinity, thus likely to promote pollination success for the available pollen, leading to higher seed production than anticipated. This is in contrast to an exceptionally low seed to ovule ratio at Fowlers Camp, a sheltered meadow with weak tidal current, and exceptionally high flowering in elevated salinities. No reproductive data were collected for Carnac Island; however, large numbers of viable fruit are observed annually. Carnac Island appears to be a special case where high clonal diversity, parental inbreeding, complete outcrossing and high proportion of full sibs are consistent with pollination and recruitment occurring within this highly sheltered meadow (as proposed in Sinclair *et al.* 2014a).

The magnitude of pollen limitation observed in natural populations depends on both historical constraints and contemporary ecological factors (Knight *et al.* 2005). Pollen limitation has been reported in several seagrass genera *Phyllospadix* spp. (Shelton 2008; Buckel *et al.* 2012), *Thalassia testudinum* (Van Tussenbroek *et al.* 2016b) and *Zostera* spp. (Reusch 2003; Van Tussenbroek *et al.* 2016a), where there is dominance of a few large clones and/or high spatial and temporal heterogeneity in flowering. Stigmas on flowers in Guischenault and Red Hill Bay may be exposed to a large amount of local pollen through strong tidal water movement, but outcrossing rates were lower because pollen was produced by a few clones, leading to selfing and/or apomixis.

Levin (2012) and Breed *et al.* (2015) highlight declining outcrossing rates in range edge and disturbed environments as a result of environmental changes. Increased selfing may be advantageous in range edge populations due to the possible advantages of reproductive assurance and through maintaining locally adapted genotypes (Arnaud-Haond *et al.* 2006; Levin 2012), despite the risks of increased mutational load that reduces fitness (Willi *et al.* 2018). Substantial increases in self-fertilization rates may also occur via plastic responses to stress (Levin 2012). An experimental study of an annual succulent halophyte *Cakile maritima* reported significant decline in plant biomass, as well as the number and size of fruit, with elevated salinity (Debez *et al.* 2004). The accumulation of Na^+^ and Cl^−^ in pollen and stigmas is known to be strongly implied in salt-induced sterility in rice (*Oryza sativa*, Khatun *et al.* 1995). Sparse flowering records in *P. australis* with low seed to ovule ratio and no viable fruit recovered from meadows growing at elevated salinities (>38 PSU) are consistent with this finding. Pseudoviviparous plantlets in unfertilized inflorescences have also been observed following lower-than-usual water temperature and complete seed abortion when plants were under thermal stress from an extreme marine heat wave (Sinclair *et al.* 2016b). This suggests a trade-off between sexual and asexual reproduction which may also be driven by both salinity and temperature (e.g. Salter *et al.* 2010). However, additional information on the spatial extent of fecund meadows is required to interpret ecological comparisons of sexual reproduction.

### Implications for long-term resilience and restoration

Strongly clonal species are known to survive for very long periods of time (de Witte and Stocklin 2010). Individual clones can persist thousands of years, surviving through significant climatic events, and essentially buffering species or populations against short-term or localized stress (e.g. Reusch *et al.* 1999; May *et al.* 2009; Arnaud-Haond *et al.* 2012). Surviving such events requires the genetic capacity to adapt and/or the propensity to shift geographical ranges (i.e. associated with sea level change). Honnay and Bossuyt (2005) argue that prolonged and nearly exclusive clonal growth through environmental suppression of sexual reproduction can ultimately lead to local sexual extinction and to monoclonal populations, with significant consequences for population viability.

Shark Bay was impacted by an extreme marine heatwave in 2010/11 which caused significant loss of seagrass (Fraser *et al.* 2014; Thomson *et al.* 2015) and sexual reproductive failure in *P. australis* (Sinclair *et al.* 2016b). A recent review of the impacts of this heatwave showed it has taken 6 years to observe natural recovery of shoot density (Kendrick *et al.* 2019). However, this recovery is likely driven through rhizome expansion rather than sexual recruitment (Kendrick *et al.* 2019), as seed production is poor and patchy.

Conservation and mitigation of disturbance have typically been the first line of defence for seagrass loss, but ecological restoration is becoming increasingly necessary in a rapidly changing environment (Statton *et al.* 2018). It is potentially a more effective management strategy where seagrass habitat has been recently lost or heavily impacted and sexual reproduction is sporadic, as natural recruitment events are rare. Tackling restoration in warming range edge populations across environmental gradients may present additional challenges. Sexton *et al.* (2011) manipulated patterns of gene flow in an annual plant to experimentally show that offspring fitness improved with outcrossing, but that lifetime reproductive success only increased significantly when pollen originated from other warm edge populations. They emphasized the overlooked importance of gene flow among populations occurring near the same range edge, highlighting the potential for prescriptive gene flow as a conservation/restoration option. Restoration of marine ecosystems will benefit from vigorous debates around the use of local (maintain local adaptation) versus non-local (mitigate against climate change) plant material (Breed *et al.* 2018). Other alternatives include the use of population genomics to understand the genetic basis of adaptation to inform seed sourcing (Breed *et al.* 2019).

Seagrasses have persisted for thousands of years through multiple climate cycles, with no recent evidence of latitudinal range contraction in *P. australis*. The range edge meadows of Shark Bay have retained (neutral) genetic diversity and the ability to reproduce sexually (albeit lower). The use of vegetative (clonal) and seed material from multiple *P. australis* meadows across Shark Bay to assist recovery may artificially enhance meadow diversity and outcrossing rates for better quality seed production in the future. Ongoing research into the role of adaptation, acclimation and plasticity in range edge meadows may shed light on how these meadows with reduced sexual reproduction and outcrossing rates may overcome additional challenges across a salinity gradient under changing climates.

## Supporting Information

The following additional information is available in the online version of this article—

**Table S1.** Seven labelled polymorphic microsatellite loci isolated from *Posidonia australis*.

**Table S2.** Allele frequencies for all genotyped samples by meadow and tissue type (maternal shoots and embryos). Based on tri-allelic genotypes and calculated in Genodive.

**Table S3.** Genetic differentiation as estimated using *F*_ST_ (above diagonal) and *P*-values (below diagonal) between all pairs of maternal shoots and embryo ‘populations’.

plaa038_suppl_Supplementary_Table_S1

plaa038_suppl_Supplementary_Table_S2

plaa038_suppl_Supplementary_Table_S3

## Data Availability

All raw microsatellite genotype data are available from the UWA Research Repository at: 10.26182/5f0bfbc877c60.
